# Analysis of the Adsorption-Release Isotherms of Pentaethylenehexamine-Modified Sorbents for Rare Earth Elements (Y, Nd, La)

**DOI:** 10.3390/polym14235063

**Published:** 2022-11-22

**Authors:** Matteo Di Virgilio, Saverio Latorrata, Cinzia Cristiani, Giovanni Dotelli

**Affiliations:** Department of Chemistry, Materials and Chemical Engineering “Giulio Natta”, Politecnico di Milano, Piazza Leonardo da Vinci 32, 20133 Milano, Italy

**Keywords:** WEEE, activated carbon, pentaethylenehexamine, rare earth elements, adsorption, release, Langmuir isotherm, Freundlich isotherm, linear regression

## Abstract

Waste from electrical and electronic equipment (WEEE) is constantly increasing in quantity and becoming more and more heterogeneous as technology is rapidly advancing. The negative impacts it has on human and environment safety, and its richness in valuable rare earth elements (REEs), are accelerating the necessity of innovative methods for recycling and recovery processes. The aim of this work is to comprehend the adsorption and release mechanisms of two different solid sorbents, activated carbon (AC) and its pentaethylenehexamine (PEHA)-modified derivative (MAC), which were deemed adequate for the treatment of REEs deriving from WEEE. Experimental data from adsorption and release tests, performed on synthetic mono-ionic solutions of yttrium, neodymium, and lanthanum, were modelled via linear regression to understand the better prediction between the Langmuir and the Freundlich isotherms for each REE-sorbent couple. The parameters extrapolated from the mathematical modelling were useful to gain an a priori knowledge of the REEs–sorbents interactions. Intraparticle diffusion was the main adsorption mechanism for AC. PEHA contributed to adsorption by means of coordination on amino groups. Release was based on protons fostering both a cation exchange mechanism and protonation. The investigated materials confirmed their potential suitability to be employed in real processes on WEEE at the industrial level.

## 1. Introduction

Waste from electrical and electronic equipment (WEEE) is defined as all those electrical and electronic components that have reached their end-of-life or have failed to enter the market due to defectiveness issues [1,2]. Nowadays, the quantity and heterogeneity of WEEE is continuously increasing as a direct consequence of the fast technological innovation the world is facing [2,3]. In parallel, the intrinsic dangerousness of WEEE due to the negative effects it can have on human health and environment is becoming more and more addressed within the scientific community [3,4]. Several scholars are indeed focusing their work on the development of suitable methods for recycling WEEE in an eco-friendly and cost-effective fashion, i.e., the so-called urban mining [4,5,6]. Special emphasis is being given to the exploitation of such waste streams as a secondary source of valuable raw materials [4,7].

Among the many critical metals which WEEE are rich in [8,9], rare earth elements (REEs) occupy a prime position in terms of importance and necessity [10]. REEs are a sub-group of the rare metals (RMs) category. The sub-group is composed of 15 lanthanides, plus scandium and yttrium [9,10]. Their unique physicochemical properties make them highly demanded. Nonetheless, their availability is restricted to few and rapidly depleting Chinese reserves, in which mining is often challenging [6,11,12]. The European Commission is prioritizing the disengaging from this compulsory dependence by directing the scientific research toward innovative solutions for the recovery of critical REEs from WEEE [7,10,11,13,14,15]. In this way, both socio-economic benefits, due to the possibility of diversifying REEs supply, and environmental benefits, due to the minimization of the disposal of harmful wastes in soil and water, could be achieved [10,14,16,17].

Many different techniques were studied for such purpose, including chemical precipitation, adsorption, crystallization, ion exchange, coagulation, flocculation, and electrodialysis [18]. Solid/liquid adsorption can be considered as one of the preferred methods for the capture of REEs in cationic form contained in mixtures of dissolved WEEE, thanks to its overall simplicity, economic convenience, flexibility, and reversibility [14,17,19,20,21]. Moreover, the possibility to employ suitable materials from natural sources makes solid/liquid adsorption a green answer to the problem of REEs recovery. Clay-based materials [13,22,23,24,25,26,27,28] and carbon-based materials [29,30,31,32,33,34,35], either in a pristine form or appropriately modified, are typical exponents of the category of natural sorbents, and have been broadly discussed in literature.

However, the development of novel typologies of solid sorbents is covering a significant portion of the efforts revolving around this topic, since the requirements of high efficiency, capability to perform well in case of low-concentration solutions, and selectivity toward specific REEs are becoming urgent [6,12,15,36].

The authors’ research group dealt with the investigation of innovative sorbents such as mineral clays [17,37], organoclays [37,38] and activated carbon [20,39] in multiple works. Activated carbon (AC) is widely used for the adsorption of metallic elements from aqueous solutions due to its inherent porosity, high specific surface area, peculiar surface chemistry, and cheapness [31,35,40]. Its modification with pentaethylenehexamine (PEHA) proved to be appropriate to increase the AC capability to adsorb metal cations. By means of the application of appropriate experimental conditions, PEHA can be intercalated in its neutral form, allowing the preservation of the amino groups that actively participate to the adsorption phenomenon. Furthermore, the combination of AC and PEHA also guarantees an improved desorption tendency, which is fundamental for the regeneration of the solid sorbent aimed at reusing it as many times as possible, and thus in a sustainable way [20].

Within such context, this work intends to be a step further toward the comprehension of the adsorption and release mechanisms of three REEs, namely yttrium (Y), neodymium (Nd), and lanthanum (La), on both AC and its modified derivative (MAC). In previous works, only the capture and release capability of both pristine and PEHA-modified clays and activated carbons (AC) towards Ni and La were evaluated [20,39], and AC and MAC were characterized morphologically and micro-structurally upon La adsorption [20], which was taken as representative of REEs. Moreover, only a preliminary thermodynamic analysis on La capture was discussed. In the present work, experiments with Y, Nd, and La on both AC and MAC were performed to deeply analyze isotherms and to model adsorption/release behavior with the goal to evidence not only capture and release capability, but also to find out possible selectivity effects of sorbents, as well as ion-sorbent interaction mechanisms. A trustworthy prediction of the cation–sorbent interactions during the adsorption and the release processes would be useful for the implementation and management of these materials at an industrial scale. Starting from REEs mono-ionic solutions at different concentrations (10–200 mmol L^−1^), adsorption and release experiments were carried out for both materials, in order to clarify the corresponding mechanisms and how the presence of PEHA influenced them. To accomplish such purpose, a mathematical modelling of the experimental data was performed by exploiting the Langmuir and the Freundlich isotherms, which are the most used non-linear predictive models for the adsorption-release behavior of solid sorbents [19,41,42,43,44,45,46]. Linear regressions in different forms were adopted, both to understand which model was better and to extrapolate the most reliable parameters for each specific REE-sorbent system. The performances of the studied materials in terms of adsorption and release efficiencies were compared as well, to verify the positive effects of the intercalation of PEHA in the AC matrix. Adsorption of cations onto AC was not influenced by the pore size of sorbents, and the main mechanism was supposed to be intraparticle diffusion [47], whereas the electron lone pairs of nitrogen atoms of MAC led to a mechanism mainly based on coordination. In the end, the results were interpreted to hypothesize a hierarchy of REE affinity with respect to each sorbent, in the perspective of the processing of complex multi-ionic solutions and of real WEEE streams.

## 2. Materials and Methods

### 2.1. Materials

The solid sorbents employed in this work were activated carbon (AC) and a modified version of the same material, identified as modified activated carbon (MAC), which were fully characterized in [20]. AC was supplied by Torchiani S.r.l. (Brescia, Italy) in a powdered form. It was characterized by a particle size lower than 120 μm (mesh of 99.8%), a specific surface area (SSA) of 575 m^2^ g^−1^ measured by BET analysis, a density of 600 kg m^−3^, moisture and ash contents of 10 wt % and 10–15 wt %, respectively, and a pore volume of 0.39 cm^3^ g^−1^.

The preparation of MAC involved the intercalation of pentaethylenehexamine (PEHA), acquired from Sigma Aldrich (St. Louis, MO, USA). This linear low molecular weight molecule, whose brute formula is C_10_H_28_N_6_, has a molecular mass of 232 g mol^−1^ and a density of 9600 kg m^−3^. According to the procedure discussed in [20,38], AC and an aqueous PEHA solution (pH = 11) were stirred at 500 rpm in a jacketed reactor at a controlled temperature of 30 °C for 90 min. The proposed procedure allowed the intercalation of PEHA in its neutral form, i.e., absence of ion exchange. For the functionalized material, a lower specific surface area (69 m^2^ g^−1^) and a decreased pore volume (0.09 cm^3^ g^−1^) were observed.

Mono-ionic aqueous solutions of the three REEs of interest were prepared through dissolution of Y(NO_3_)_3_∙6H_2_O, Nd(NO_3_)_3_∙6H_2_O, and La(NO_3_)_3_∙6H_2_O (purity of 99.9%, from Sigma Aldrich, St. Louis, MO, USA) in deionized water. For each REE, solutions with concentration of 10, 19, 40, 60, 80, 100, and 200 mmol L^−1^ were investigated in adsorption and release experiments for both AC and MAC.

### 2.2. Methods

**Figure 1:**
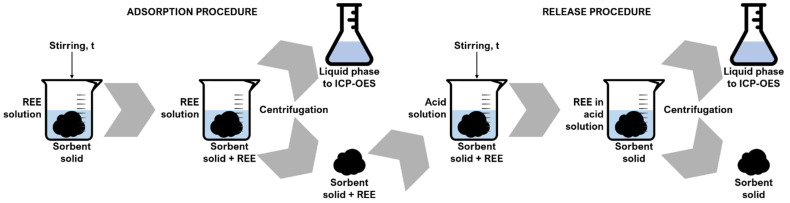
Adsorption and release experimental procedures.

Adsorption and release experiments were performed according to the procedure disclosed in [5,38], sketched in Figure 1.

Briefly, 2 g of solid sorbent were contacted with the mono-ionic aqueous solutions of each REE under continuous agitation (1500 rpm), at room temperature and atmospheric pressure, for a time varying between 10 min and 24 h to reach the equilibrium conditions. Afterwards, centrifugation at 3500 rpm was carried out via the RotoFix 32A centrifuge (Hettich Italia S.r.l., Milan, Italy) to separate the solid and the liquid phases.

Release of the adsorbates was performed by immersing 1.3 g of solid sorbent in a strongly acidic HNO_3_ (Sigma Aldrich, St. Louis, MO, USA) solution (pH = 1) and by continuously stirring (1500 rpm) at room temperature. The liquid phases were again separated by centrifugation in the abovementioned conditions. Post-adsorption and post-release experiments’ liquid phases were analyzed via inductively coupled plasma optical emission spectroscopy (ICP-OES) by employing the spectrometer Optima 2000 DV (PerkinElmer, Inc., Waltham, MA, USA), in order to determine the cationic concentrations of dissolved REEs [48,49]. In a previous paper, sorbents reusability has been demonstrated at the lab scale via release experiments, and up to four cycles of adsorption/release processes were performed without any change in performances [20]. In this paper, the release step was studied mainly to evaluate possible sorbent selectivity towards the different metals. This point, indeed, is of paramount importance considering that sorbent selectivity is still an open point. Knowledge on the selectivity behavior during release may allow the possibility to control the process, and thus REs separation effectively.

### 2.3. Mathematical Approach

By defining, for each investigated solution, the initial concentration as C_0_ (mmol L^−1^) and the equilibrium concentration measured via ICP-OES as C_eq_ (mmol L^−1^), the specific quantity of cations adsorbed on the solid (Q_eq_, mmol g_sol_^−1^) was extrapolated via Equation (1):

(1) $$Q_{\mathrm{eq}}=\frac{\left(C_{0}-C_{\mathrm{eq}}\right)\cdot V}{m}$$

where V is the solution volume (0.05 L), and m is the mass of the solid sorbent (2 g). The corresponding adsorption efficiency (η_ads_, %) was computed according to Equation (2):

(2) $$\eta_{\mathrm{ads}}=\frac{\left(C_{0}-C_{\mathrm{eq}}\right)}{C_{0}}\cdot 100$$

The quantity of released cations per unit mass of the solid (Q_rel_, mmol g_sol_^−1^) was calculated via Equation (3), in which C_rel_ (mmol L^−1^) is the cation concentration in solution after the release experiment, evaluated via ICP-OES, V is the solution volume (0.05 L), and m is the mass of the solid sorbent (1.3 g):

(3) $$Q_{\mathrm{rel}}=\frac{C_{\mathrm{rel}}\cdot V}{m}$$

Consequently, the quantity of residual cations on the solid (Q_res_, mmol g_sol_^−1^) was determined via Equation (4):

(4) $$Q_{\mathrm{res}}=Q_{\mathrm{eq}}-Q_{\mathrm{rel}}$$

The release efficiency (η_rel_, %) was computed through Equation (5):

(5) $$\eta_{\mathrm{rel}}=\frac{Q_{\mathrm{rel}}}{Q_{\mathrm{eq}}}\cdot 100$$

Modelling of the adsorption and release behavior of AC and MAC relied on the Langmuir and the Freundlich isotherms, which are the most widely used two-parameters models for the analysis of these phenomena. Their generic constitutive expressions are reported as Equations (6) and (7), respectively:

(6) $${Q=q}_{m}\cdot \frac{K_{L}\cdot C}{{1+K}_{L}\cdot C}$$

(7) $${Q=K}_{F}\cdot C^{\frac{1}{n}}$$

The term Q is Q_eq_ for adsorption and Q_res_ for release, whereas C is C_eq_ for adsorption and C_rel_ for release. Concerning the Langmuir isotherm, q_m_ (mmol g^−1^) represents the maximum adsorption or release capacity of one single sorbent layer and K_L_ (L mmol^−1^) is the Langmuir constant, related to the free adsorption or release energy. In the Freundlich isotherm, the dimensionless parameter n gives information on the adsorption or release tendency of the sorbent, while K_F_ (L^1/n^ mmol^(n–1)/n^ g^−1^) is the Freundlich constant for heterogeneous sites.

The extrapolation of the parameters for both models was performed via linear regression, by exploiting three different linearization forms for the Langmuir model [42,44] and one conventional linearization for the Freundlich model [42,43]. Table 1 gathers all these linear forms, the corresponding plots, and the extrapolated parameters.

## 3. Results

Since the experimental conditions explained in Section 2.2 preserved the oxidation state of all the involved cations, they are always cited in the following Sections without specifying their charge, i.e., in the form Y, Nd, and La. For the sake of clarity, the results for AC and MAC are presented in two separate and coherently entitled Sections. In each of them, the modelling of adsorption and release behavior of Y, Nd, and La is individually elucidated and interpreted.

All adsorption experimental data of Y, Nd, and La, i.e., initial concentration (C_0_), equilibrium concentration (C_eq_), and corresponding specific quantity of cations adsorbed on the solid (Q_eq_), are summarized in Tables S1 and S2 (Supplementary Information) for AC and MAC, respectively. Analogously, all release experimental data of the three REEs, i.e., concentration of released cations (C_rel_) in solution, quantity of released cations per unit mass of the solid (Q_rel_), and quantity of residual cations on the solid (Q_res_), are reported in Tables S3 and S4 for AC and MAC, respectively. These Tables are not quoted in the following Sections anymore.

### 3.1. Activated Carbon

#### 3.1.1. Adsorption

**Figure 2:**
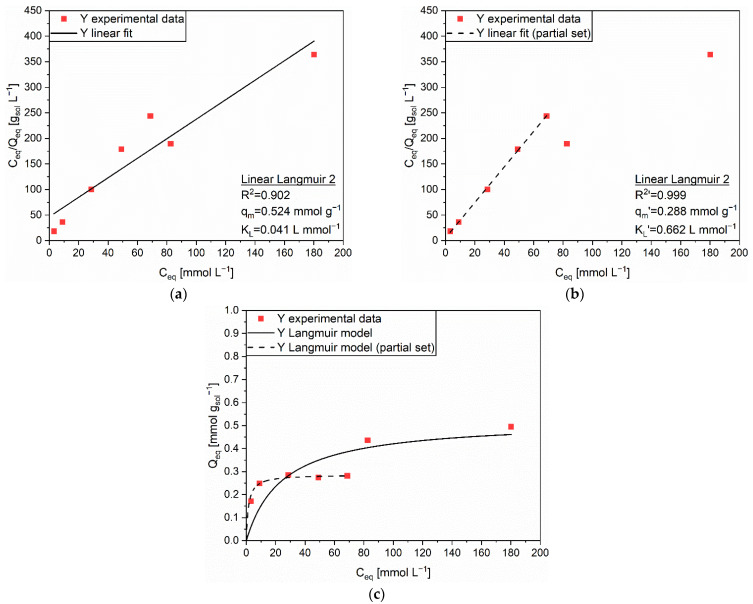
(**a**) Linear regression using Linear Langmuir 2 (full line) and corresponding extrapolated parameters; (**b**) Linear regression using Linear Langmuir 2 on the partial set (dashed line) and corresponding extrapolated parameters; (**c**) Comparison of experimental data and predicted data (full and dashed lines) with the Langmuir isotherm for the adsorption of Y (■) on AC.

The adsorption behavior of Y on AC complied with the Langmuir isotherm. The best fitting was achieved by using the Linear Langmuir 2 equation, whose correlation coefficient (R^2^ = 0.902) was the highest among the ones provided by the different linear regressions on the experimental data set (Table S5) The correspondence was quite satisfactory, as displayed in Figure 2a. Nevertheless, the Q_eq_ values determined for the highest equilibrium concentrations, corresponding to initial concentrations of 100 and 200 mmol L^−1^, seemed to deviate from the other ones. A possible explanation was the attainment of an initial saturation of a single layer of adsorption sites for Q_eq_ ≈ 0.3 mmol g_sol_^−1^, and the subsequent occurrence of a mechanism change fostered by the activation of new adsorption sites when the sorbent was subjected to initial concentrations equal to or higher than 100 mmol L^−1^. This two-steps process could be associated to the distribution of pore diameters in AC, which was hypothesized to be bi-modal [20]. The mathematical analysis was repeated by excluding the divergent data, allowing to reach a much more reliable result (R^2′^ = 0.999, Figure 2b). With the new extrapolated parameters, q_m_’ = 0.288 mmol g^−1^ and K_L_’ = 0.622 L mmol^−1^, the prediction of the Langmuir model (Figure 2c, dashed line) appeared to be excellent.

**Figure 3:**
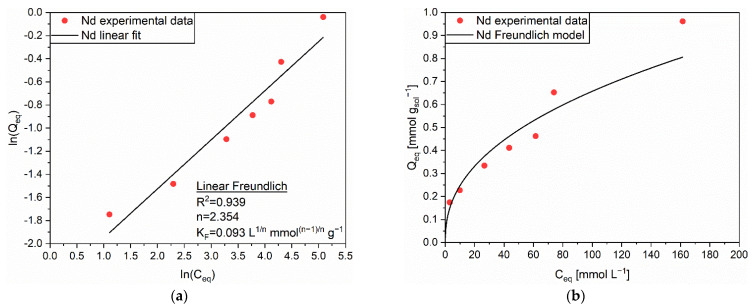
(**a**) Linear regression using Linear Freundlich (full line) and corresponding extrapolated parameters; (**b**) Comparison of experimental data and predicted data (full line) with the Freundlich isotherm for the adsorption of Nd (●) on AC.

The Freundlich isotherm was identified as the most reliable one to explain the adsorption behavior of Nd on AC, thanks to a R^2^ value of 0.939 (Figure 3a). The heterogeneous nature of the complex AC surface was indicated as the main reason for such outcome [40]. Differently from Y, Nd appeared to follow a single-step mechanism according to which the intraparticle diffusion of cations was not affected by the different pore dimensions of the solid sorbent. Regarding to this, a slightly better affinity between Nd and AC with respect to Y and AC could be presumed. The corresponding extrapolated parameters, n = 2.354 and K_F_ = 0.093 L^1/n^ mmol^(n–1)/n^ g^−1^, permitted a sufficiently accurate prevision of the data obtained from the adsorption experiments, as can be appreciated from Figure 3b.

**Figure 4:**
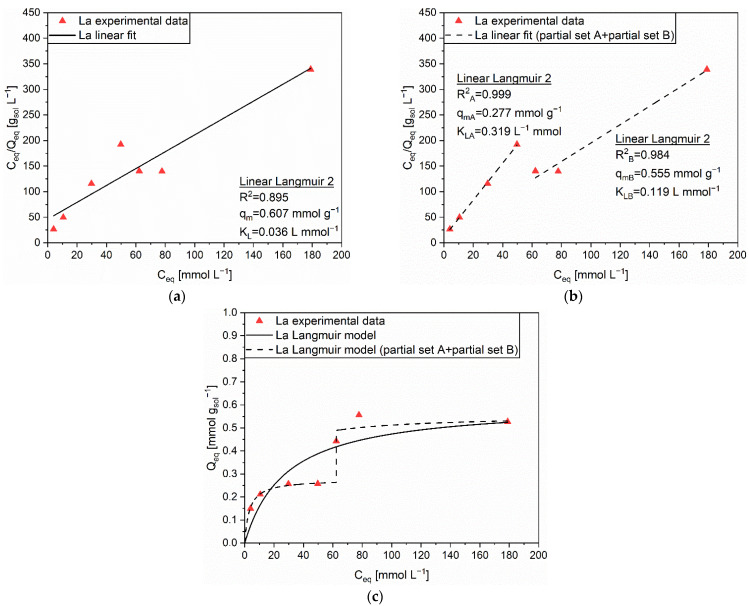
(**a**) Linear regression using Linear Langmuir 2 (full line) and corresponding extrapolated parameters; (**b**) Linear regression using Linear Langmuir 2 on partial sets A and B (dashed line) and corresponding extrapolated parameters; (**c**) Comparison of experimental data and predicted data (full and dashed lines) with the Langmuir isotherm for the adsorption of La (▲) on AC.

The Langmuir isotherm was chosen for the modelling of the adsorption behavior of La on AC. However, the corresponding R^2^ of 0.895 (Figure 4a) achieved with the Linear Langmuir 2 equation, and the visible increase of cations adsorbed on the solid above equilibrium concentrations higher than 50 mmol L^−1^, suggested the possibility of a mechanism variation above a threshold initial concentration, which was recognized as 60 mmol L^−1^. Consequently, two different sets of experimental data were separately investigated. Set A accounted for four C_eq_ values lower than 50 mmol L^−1^, while set B considered three C_eq_ values higher than 50 mmol L^−1^. In both cases, the Linear Langmuir 2 equation provided the most reliable fittings (Figure 4b), with correlation coefficients equal to R^2^_A_ = 0.999 and R^2^_B_ = 0.984, respectively. With the extrapolated parameters q_mA_ = 0.277 mmol g^−1^, K_LA_ = 0.319 L mmol^−1^, q_mB_ = 0.555 mmol g^−1^, and K_LB_ = 0.119 L mmol^−1^, the prediction of the La adsorption behavior on AC seemed to be highly precise (Figure 4c, dashed line). Once again, a two-steps process characterized by an activation of different adsorption sites, able to accommodate a new single layer of adsorbates, was deemed as a possible explanation for these outcomes.

#### 3.1.2. Release

**Figure 5:**
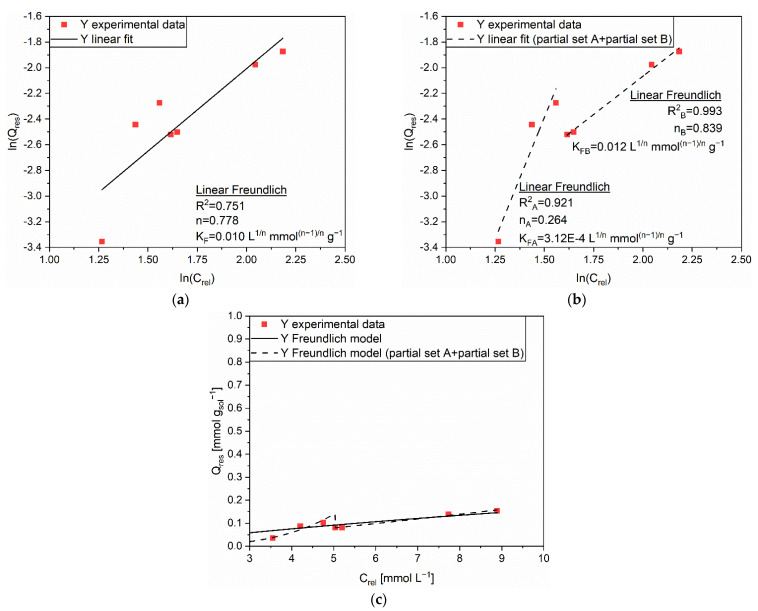
(**a**) Linear regression using Linear Freundlich (full line) and corresponding extrapolated parameters; (**b**) Linear regression using Linear Freundlich on partial sets A and B (dashed line) and corresponding extrapolated parameters; (**c**) Comparison of experimental data and predicted data (full and dashed lines) with the Freundlich isotherm for the release of Y (■) from AC.

Concerning the release of Y from AC, it can be observed that the values of C_rel_ and Q_rel_ followed a quasi-linear evolution: the higher the quantity of adsorbed cations, the higher the quantity of released ones. In the first analysis, the R^2^ values obtained from the linear regressions recommended the Freundlich isotherm (R^2^ = 0.751, Figure 5a) as the best fitting for such a release experiment data set. However, the existence of a visible step in the Q_res_ trend for values higher than 100 mmol g_sol_^−1^ hinted at a possible change in the desorption mechanism. This observation complied with the previously described two-steps process for the adsorption of Y on AC. The solid sorbent appeared to release cations with a bit more difficulty when Q_eq_ approached the threshold value for the activation of new sites. Then, the release capacity underwent an increase when the adsorption of the two independent monolayers occurred. In order to better elucidate such release behavior, the experimental data were divided in set A, which considered three Q_res_ values lower than 100 mmol g_sol_^−1^, and set B that accounted for the remaining four Q_res_ values. The Freundlich isotherm produced trustworthy fittings (Figure 5b), the correlation coefficients being equal to R^2^_A_ = 0.921 and R^2^_B_ = 0.993, respectively. The correspondence between the experimental data and the predicted data with the extrapolated coefficients (n_A_ = 0.264, K_FA_ = 3.12E–4 L^1/n^ mmol^(n–1)/n^ g^−1^, n_B_ = 0.839, and K_FB_ = 0.012 L^1/n^ mmol^(n–1)/n^ g^−1^), depicted in Figure 5c, was considered satisfactory.

**Figure 6:**
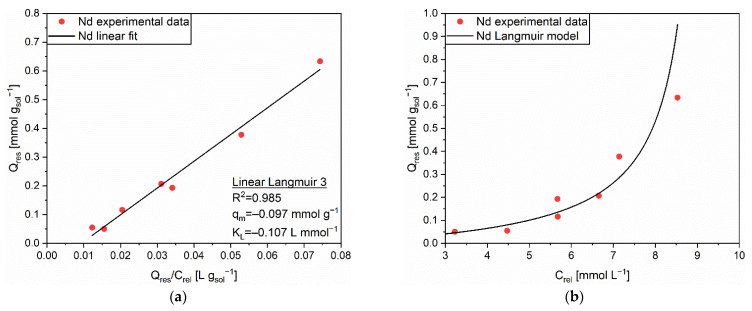
(**a**) Linear regression using Linear Langmuir 3 (full line) and corresponding extrapolated parameters; (**b**) Comparison of experimental data and predicted data (full line) with the Langmuir isotherm for the release of Nd (●) from AC.

Differently from the case of adsorption, which obeyed the Freundlich isotherm, the release of Nd from AC was better described by the Langmuir isotherm, since the best fitting was achieved by exploiting the Linear Langmuir 3 equation (R^2^ = 0.985, Figure 6a). This outcome could be explained by the action of the highly acidic environment in which the release experiment was performed. The high concentration of protons promoted a fast cation exchange mechanism, through which the bounded Nd cations were homogeneously and simultaneously released without mutually interacting. In general, an increase of the release capacity was appreciated for a higher amount of adsorbed cations. In Figure 6b, the prediction of the Langmuir model with the extrapolated parameters, q_m_ = −0.097 mmol g^−1^ and K_L_ = −0.107 L mmol^−1^, was quite precise. The only exception was the release at the highest quantity of adsorbates, for which a small divergence between the experimental and the predicted data was identified.

**Figure 7:**
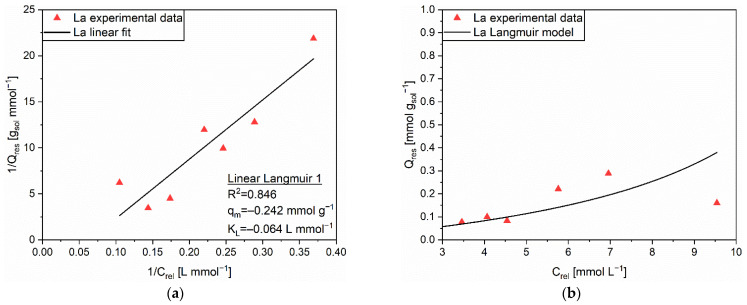
(**a**) Linear regression using Linear Langmuir 1 (full line) and corresponding extrapolated parameters; (**b**) Comparison of experimental data and predicted data (full line) with the Langmuir isotherm for the release of La (▲) from AC.

From the values of the correlation coefficients R^2^ (Table S6), the Langmuir isotherm was evaluated as the most reliable to explain the release process of La from AC. Figure 7a shows the corresponding regression, which relied on the Linear Langmuir 1 equation. The R^2^ value was 0.846, whereas the extrapolated coefficients were q_m_ = −0.242 mmol g^−1^ and K_L_ = −0.064 L mmol^−1^, respectively. Similar to the case of Nd, the highly acidic environment typical of the release experiment was deemed as the main factor triggering the release of La cations via a homogeneous exchange mechanism. The prevision guaranteed by the Langmuir isotherm appeared to sufficiently fulfill the experimental data (Figure 7b), although a trend discrepancy can be pinpointed at the highest C_rel_ (hence, Q_eq_) value. A direct influence of the two-steps adsorption mechanism, which involved larger Q_eq_ values, was speculated as a plausible justification, since the C_rel_ and Q_rel_ values proved quasi-linear once again.

### 3.2. Modified Activated Carbon

#### 3.2.1. Adsorption

**Figure 8:**
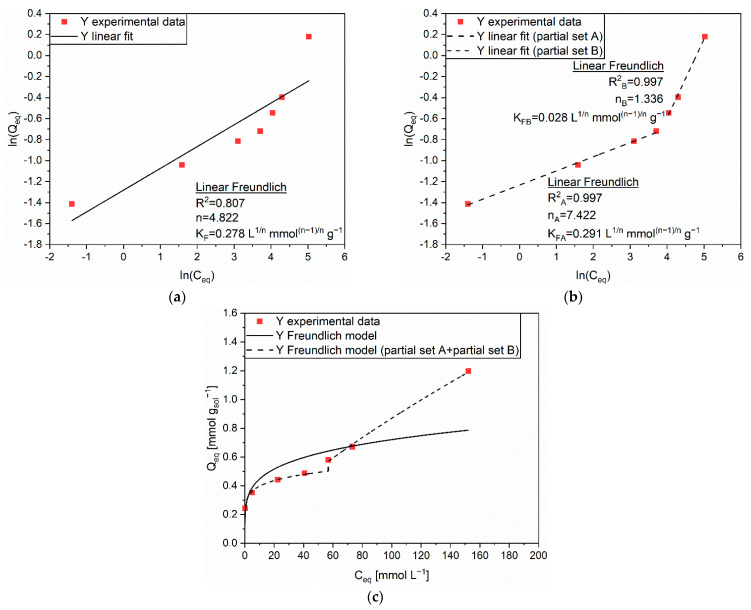
(**a**) Linear regression using Linear Freundlich (full line) and corresponding extrapolated parameters; (**b**) Linear regression using Linear Freundlich on partial sets A and B (dashed line) and corresponding extrapolated parameters; (**c**) Comparison of experimental data and predicted data (full and dashed lines) with the Freundlich isotherm for the adsorption of Y (■) on MAC.

Due to the linear regression outcomes in Table S7, the Freundlich isotherm was recommended for the modelling of the adsorption mechanism of Y on MAC. Figure 8a displays the linear trend associated to the experimental data (R^2^ = 0.807), and the corresponding extrapolated parameters (n = 4.822 and K_F_ = 0.278 L^1/n^ mmol^(n–1)/n^ g^−1^). Nonetheless, the model was judged as not sufficiently accurate, since the presence of PEHA strongly influenced the adsorption capability of the solid matrix. As discussed in [20], the heterogeneity pointed out by the Freundlich model could be elucidated by a complex combination of different mechanisms. While intraparticle diffusion in the pores still represented the main contribution due to the solid sorbent at lower initial concentrations, PEHA actively participated to adsorption by coordinating cations through its multiple amino groups (both –NH and –NH_2_), rich in electron lone pairs on the nitrogen atoms. Considering this, two sets of experimental data were analyzed individually, the first one accounting for C_0_ values lower than 70 mmol L^−1^, the second one for C_0_ values higher than 70 mmol L^−1^. The linear regressions provided highly accurate results, with R^2^ values of 0.997 for both sets (Figure 8b). The corresponding extrapolated parameters were n_A_ = 7.442 and K_FA_ = 0.291 L^1/n^ mmol^(n–1)/n^ g^−1^, and n_B_ = 1.336 and K_FB_ = 0.028 L^1/n^ mmol^(n–1)/n^ g^−1^, respectively. In Figure 8c, the comparison between the modelled data and the experimental data confirmed the validity of the hypothesized combined process, since the partial Freundlich isotherms (dashed line) are practically superimposed to the experimental data.

**Figure 9:**
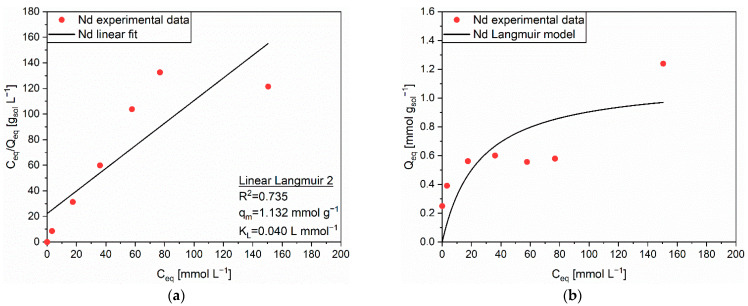
(**a**) Linear regression using Linear Langmuir 2 (full line) and corresponding extrapolated parameters; (**b**) Comparison of experimental data and predicted data (full line) with the Langmuir isotherm for the adsorption of Nd (●) on MAC.

Moving to the adsorption of Nd on MAC, the Langmuir isotherm was chosen to describe the adsorption mechanism, due to a R^2^ of 0.735 obtained from the Linear Langmuir 2 equation (Figure 9a). However, such a result evidenced the possibility of a mechanism variation above C_eq_ of 80 mmol L^−1^, corroborated by the existence of a step in the corresponding Q_eq_ values and a scarce prediction suitability of the model (Figure 9b). PEHA appeared to slightly alter the previously discussed affinity between Nd and AC up to an initial concentration of 100 mmol L^−1^, inasmuch the quantity of adsorbates reached a plateau at Q_eq_ ≈ 0.6 mmol g_sol_^−1^. Most likely, the steric hindrance of the PEHA chains on the AC surface had a detrimental effect on the Nd intraparticle diffusion inside the pores. The situation changed at the highest initial concentration (200 mmol L^−1^), since PEHA was ascribed as the main affecting factor for the steep increase in adsorbed cations (Q_eq_ = 1.239 mmol g_sol_^−1^). The coordination mechanism on the amino groups was supposed to become more pronounced in conditions of high initial cations concentration.

**Figure 10:**
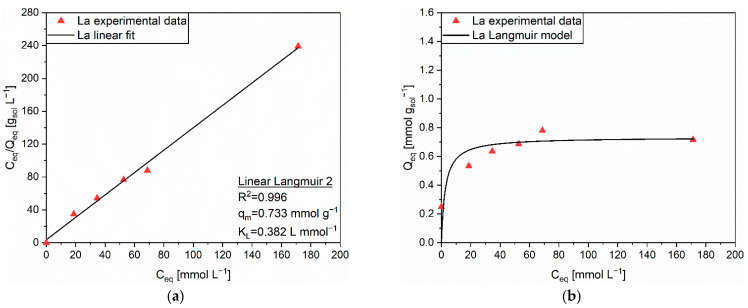
(**a**) Linear regression using Linear Langmuir 2 (full line) and corresponding extrapolated parameters; (**b**) Comparison of experimental data and predicted data (full line) with the Langmuir isotherm for the adsorption of La (▲) on MAC.

The adsorption behavior of La on MAC complied with the Langmuir isotherm, according to the results of the linear regressions (Table S7). Figure 10a highlights the linear fitting corresponding to the Linear Langmuir 2 equation, whose R^2^ was equal to 0.996. The comparison between the experimental data and the Langmuir isotherm obtained with the extrapolated parameters, q_m_ = 0.733 mmol g^−1^ and K_L_ = 0.382 L mmol^−1^, is depicted in Figure 10b. The absence of C_eq_ and Q_eq_ values for C_0_ = 19 mmol L^−1^ must be imputed to an experimental error happened during the ICP-OES measurements. MAC seemed to reach saturation of its sites, being them either conventional AC sites or the amino groups of PEHA, at Q_eq_ ≈ 0.8 mmol g_sol_^−1^. Such a trend could be symptomatic of a simultaneous participation of intraparticle diffusion in AC pores and coordination on PEHA amino groups to the overall adsorption mechanism. The identification of the contribution of the two different mechanisms was not possible from the bare modelling of the experimental data. Another possible explanation was an adsorption mechanism relying only on coordination on the PEHA chains, while AC sites were not exploited due to the steric hindrance of the intercalated molecules. In this regard, the slightly higher ionic radius of La (1.06 Å) with respect to Y (0.90 Å) and Nd (0.995 Å) [50] should be contemplated as a key factor for the hindered intraparticle diffusion.

#### 3.2.2. Release

**Figure 11:**
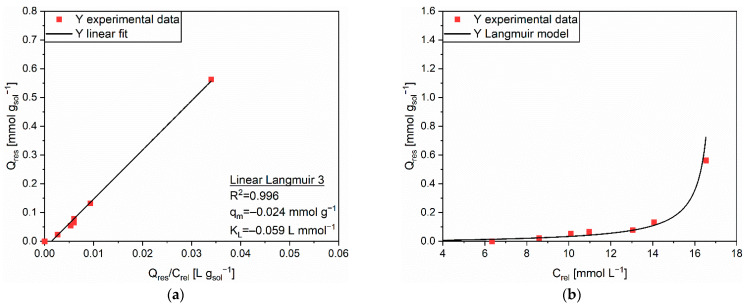
(**a**) Linear regression using Linear Langmuir 3 (full line) and corresponding extrapolated parameters; (**b**) Comparison of experimental data and predicted data (full line) with the Langmuir isotherm for the release of Y (■) from MAC.

Regarding the release of Y from MAC, the linear regression exploiting the Linear Langmuir 3 equation was evaluated as the most accurate to model the release behavior of Y from AC. The value of the correlation coefficient R^2^ was 0.996, as can be seen from Figure 11a. The prediction of the Langmuir isotherm, whose coefficients were q_m_ = −0.024 mmol g^−1^ and K_L_ = −0.059 L mmol^−1^, approximated the experimental data in a reliable fashion. Observing the trend of Q_res_ in Figure 11b, one could state the continuous improvement of the release capacity at a higher amount of adsorbed cations. This result suggested a slightly different desorption behavior between MAC and AC, for which the dominant mechanism was the cation exchange. In this case, protons from the acid solution were probably able both to substitute the adsorbates on the AC sites and to protonate the amino groups of PEHA. Such a twofold possibility for the desorption process was expected to be simultaneous. As a matter of fact, the two processes contributed to concentrations of cations released in solution which were roughly two times the ones measured for the release of Y from AC.

**Figure 12:**
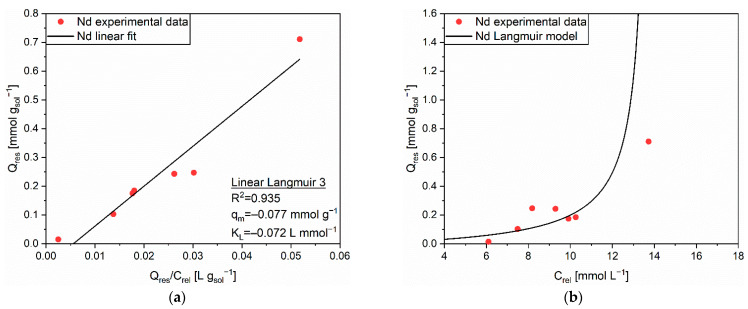
(**a**) Linear regression using Linear Langmuir 3 (full line) and corresponding extrapolated parameters; (**b**) Comparison of experimental data and predicted data (full line) with the Langmuir isotherm for the release of Nd (●) from MAC.

According to the R^2^ values of the linear regressions, the Langmuir isotherm was chosen as a predictive model for the release of Nd from the functionalized solid sorbent. The extrapolated parameters, q_m_ = −0.077 mmol g^−1^ and K_L_ = −0.072 L mmol^−1^, are shown in Figure 12a, while experimental and modelled data are plotted in Figure 12b. Notwithstanding the value of R^2^ adequately close to 1 (0.935), the correspondence between the Langmuir isotherm and the experimental data was not precise enough. In detail, two regions of discrepancy were spotted. The first region was included between released concentrations of 6 and 10 mmol L^−1^. It was characterized by almost equivalent Q_res_ values, resembling a sort of plateau of desorbed cations. An accentuated release from weakly interacting sites was hypothesized [39]. The second region involved higher C_rel_ values. It disclosed an initial reduction of Q_res_, maybe related to the adsorption on sites able to tightly bind cations, and then by a large increase up to Q_res_ ≈ 0.7 mmol g_sol_^−1^. In this last case, the quantity of adsorbed cations was so high that the desorption in the acid environment was extremely pronounced. As a consequence of these observations, the model should be conveniently refined or a new one should be adopted to correctly approximate the desorption behavior of this specific cation-sorbent system.

**Figure 13:**
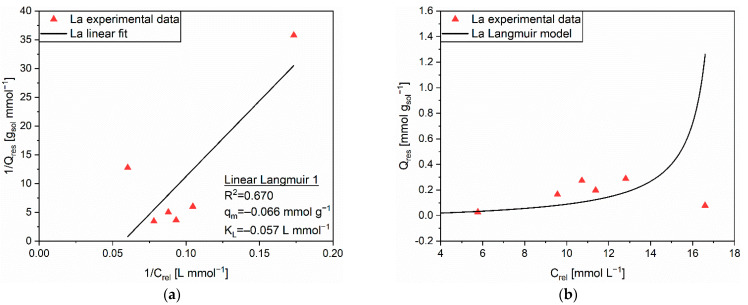
(**a**) Linear regression using Linear Langmuir 1 (full line) and corresponding extrapolated parameters; (**b**) Comparison of experimental data and predicted data (full line) with the Langmuir isotherm for the release of La (▲) from MAC.

The correlation coefficients R^2^ provided by the linear regressions on the experimental data set of the La release from MAC are summarized in Table S8. Such a desorption phenomenon appeared to obey the Langmuir isotherm model. The corresponding fitting and extrapolated parameters (q_m_ = −0.066 mmol g^−1^ and K_L_ = −0.057 L mmol^−1^) from the Linear Langmuir 1 equation (R^2^ = 0.670) are displayed in Figure 13a. The prevision provided by the Langmuir isotherm was not able to accurately approximate the experimental data, as it can be ascertained from Figure 13b. No clear trend was deduced from the Q_res_ vs. C_rel_ plot, and such an outcome was ascribed to the existence of heterogeneous electronic interactions during the desorption process [39]. Consequently, cation exchange mechanism was not regarded as sufficient to explain the release behavior of La from MAC. On the contrary, more complicated phenomena might have occurred both on the AC surface, pores, and on PEHA chains, most likely in a diverse fashion for each active site.

## 4. Discussion

**Figure 14:**
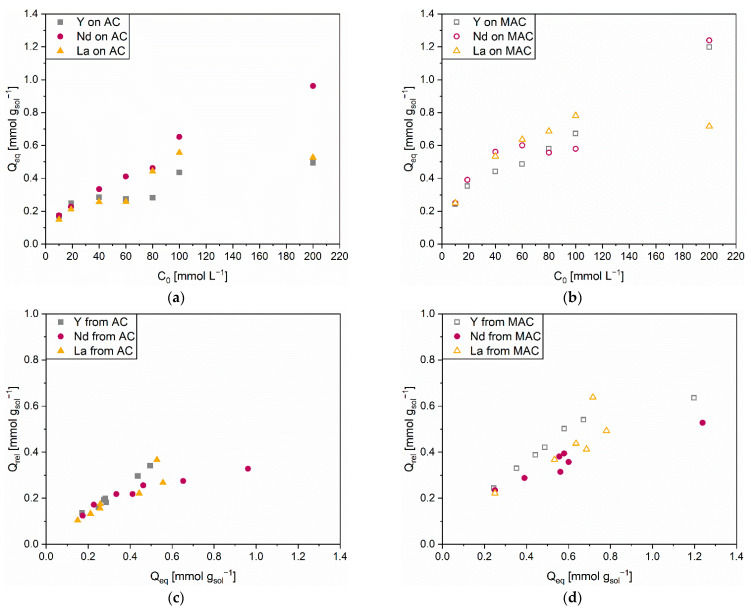
Quantity of adsorbed Y, Nd, and La cations (Q_eq_) on (**a**) AC and (**b**) MAC with respect to initial concentration (C_0_); Quantity of released Y, Nd, and La cations (Q_rel_) from (**c**) AC and (**d**) MAC with respect to quantity of adsorbed cations (Q_eq_).

Figure 14 shows a direct comparison between the adsorption and release performances of the investigated solid sorbents, AC and MAC. The first deducible information was the better adsorption tendency of MAC (Figure 14b) with respect to AC (Figure 14a), inasmuch the experimental data surveyed for the functionalized material (empty points) were above the corresponding ones measured for virgin AC (full points). This outcome was confirmed for all the investigated initial concentrations and for whichever of the considered REE. The trends of adsorption efficiency in Figure S1a,b confirm such an observation, with MAC demonstrating better performances especially at lower C_0_ values. MAC approached 100% of adsorption efficiency for Nd and La and overcame 95% for Y at C_0_ = 10 mmol L^−1^, while the corresponding values for AC were 60%–70%. Therefore, the modified sorbent ensured an almost complete adsorption of REEs for diluted metal-containing solutions.

According to the results discussed in previous works [20,23,39,51], the improvement in adsorption capacity should be ascribed to the presence of the intercalated PEHA chains. As proposed in Section 3, the amino groups (−NH, −NH_2_) operated as coordination sites thanks to their electron lone pairs on the nitrogen atoms. The actual contribution of PEHA to adsorption could be inferred by evaluating the difference in the quantity of adsorbed cations (Q_eq_) between AC and MAC. Average outcomes of 0.255 mmol g_sol_^−1^, 0.136 mmol g_sol_^−1^, and 0.235 mmol g_sol_^−1^ were calculated on the whole initial concentrations’ interval for Y, Nd, and La, respectively. Such differences roughly represented the quantity of coordinated cations on the amino groups, from which a slightly better ability of PEHA to interact with Y was comprehended. On the contrary, PEHA exerted only a minor effect on the adsorption of Nd, whose captured amount on unfunctionalized AC was already significant.

Proceeding from this, the second inferred information regarded a possible hierarchy of affinity within the investigated REE-sorbent solid couples. Concerning AC, Nd (purple full circles) resulted in the most easily adsorbed REE, especially at initial concentrations higher than 40 mmol L^−1^. Y (grey full squares), and La (orange full triangles) proved a similar inclination to adsorb on AC, albeit to a clearly lesser extent than Nd. The situation changed for MAC. As previously stated, the functionalization of the carbonaceous matrix via PEHA had a positive influence on the capacity of the solid sorbent to handle REEs. Nevertheless, the combination of intraparticle diffusion and coordination on amino groups caused a moderate loss in the hierarchy of affinity recognized for AC. No specific trends were indeed identified among the different quantities of adsorbates on MAC. While Nd was the better adsorbed cation in the range of initial concentration 20–40 mmol L^−1^ and at C_0_ = 200 mmol L^−1^, La was in the 60–100 mmol L^−1^ range before undergoing a sharp decrease of its Q_eq_ value. Moreover, the previously discussed stronger coordination of Y by means of PEHA resulted in an adsorption performance more similar to those of the other REEs. In summary, MAC proved very high efficiencies, but without preferring any of the investigated REEs.

With a view to perform experiments on multi-ionic complex solutions, these observations could be useful to determine the selectivity of these two materials toward the studied elements. Through this fundamental parameter, one would be able to wisely choose the sorbent solid able to guarantee either the best overall efficiency or a specific selectivity toward one metal rather than another when treating real WEEE streams. In other terms, there would be the possibility to tailor the process to simply purify as much as possible REEs-rich solutions coming from industrial wastes, or to recover a specific REE of interest in larger quantities.

Regarding the release process, the comparison between AC (Figure 14c) and MAC (Figure 14d) appeared less straightforward. First of all, AC released all the REE-cations in comparable amount and in a quite steady way within the whole range of initial concentrations. Release efficiencies were 70–80% for Y, 35–75% for Nd, and 50–70% for La, respectively (Figure S1c). Previous works affirmed the paramount importance of the type of eluent for an effective release of metal cations and for the regeneration of sorbent solids [15,16]. The outcomes of this work confirmed such a statement, as the release process was primarily governed by the very low pH of the HNO_3_ solution. The lower efficiency values evaluated for Nd at high adsorbate contents depended on the presumed high affinity between Nd and AC. The strong and stable interactions occurring in the AC pores caused the Nd adsorbed cations to better resist desorption due to the cation exchange mechanism.

MAC provided higher release efficiencies than AC (Figure S1d). Values of 80–100% for Y, 45–95% for Nd, and 60–90% for La, respectively, were calculated. Therefore, the intercalation of PEHA had a beneficial effect also on the desorption step. The reason for this was identified in the weaker stability of interaction between cations and amino groups, which were prone to protonation and, consequently, release. Such behavior was especially visible for Y (grey empty squares). It was indeed indicated as the most easily coordinated cation, but also proved the most released one, corroborating the observations made on the role and behavior of PEHA’s amino groups. Ultimately, MAC seemed to ensure a favored regeneration and a significant metal recovery. The possibility of reusing it should be tested by designing consecutive adsorption-release experiments, in order to determine the number of cycles it could sustain without excessively losing in terms of performance.

Future developments of this work will concern the analysis of multi-ionic solutions and, mainly, an even more in-depth mathematical modelling based on non-linear regressions. The aim would be to improve the predictive reliability of the actual adsorption-release behavior of the proposed innovative solid sorbents with respect to REEs. Since the treatment of WEEE will gain more and more importance at the industrial scale in the future, an a priori precise knowledge of how different solid sorbents interact with REEs would be really useful. In this fashion, a correct design and control of purification or recovery processes would be guaranteed.

## 5. Conclusions

This work confirmed the effectiveness of AC and its PEHA-functionalized derivative, MAC, toward the adsorption and the release of three REEs (Y, Nd, and La), representative of complex WEEE streams. The mathematical analysis of the adsorption and release outcomes from experiments on mono-ionic solutions allowed to identify the best model to describe each REE-sorbent system. In brief, the Langmuir isotherm well represented the adsorption of Y and La on AC, the adsorption of Nd and La on MAC, the release of Nd and La from AC, and the release of all the investigated REEs from MAC. Conversely, the Freundlich isotherm proved preferable to describe the adsorption of Nd on AC, the adsorption of Y on MAC, and the release of Y from AC. The functionalization by means of PEHA implicated better performance in terms of both adsorption and release, thanks to the contribution of the amino groups of PEHA. The main adsorption mechanism for AC was supposed to be intraparticle diffusion, whereas MAC was also characterized by the coordination of cations on the electron lone pairs of its nitrogen atoms. Desorption was supposed to be pH-dependent. However, this aspect should be further examined. The mechanism relied on cation-proton exchange and protonation of the amino groups fostered by the large number of protons in the eluent (HNO_3_ solution, pH = 1). The obtained results were also interpreted to recognize a plausible hierarchy of REE affinity with respect to each sorbent, in support of a real application of the studied materials at the industrial level. In this regard, AC demonstrated a higher affinity with Nd, while no specific REE preference was appreciated for MAC.

## Figures and Tables

**Table 1:** Linear forms for the Langmuir and the Freundlich models, corresponding plots, and extrapolated parameters.

**Linear Form**	**Equation**	**Plot**	**Parameters**
Linear Langmuir 1	$\frac{1}{Q}=\frac{1}{q_{m}\cdot K_{L}}\cdot \frac{1}{C}+\frac{1}{q_{m}}$	1/Q vs. 1/C	q_m_, K_L_
Linear Langmuir 2	$\frac{C}{Q}=\frac{1}{q_{m}}\cdot C+\frac{1}{q_{m}\cdot K_{L}}$	C/Q vs. C	q_m_, K_L_
Linear Langmuir 3	${Q=q}_{m}-\frac{1}{K_{L}}\cdot \frac{Q}{C}$	Q vs. Q/C	q_m_, K_L_
Linear Freundlich	$\ln\left(Q\right)=\frac{1}{n}\cdot \ln(C)+\ln\left(K_{F}\right)$	ln(Q) vs. ln(C)	n, K_F_

## Data Availability

All data are available upon reasonable request from the corresponding authors.

## Supplementary Materials

The following supporting information can be downloaded at: https://www.mdpi.com/article/10.3390/polym14235063/s1, Table S1: Initial concentrations (C_0_), equilibrium concentrations (C_eq_), and specific quantity of cations adsorbed on the solid (Q_eq_) for the adsorption of Y, Nd, and La on AC; Table S2: Initial concentrations (C_0_), equilibrium concentrations (C_eq_), and specific quantity of cations adsorbed on the solid (Q_eq_) for the adsorption of Y, Nd, and La on MAC; Table S3: Concentration of released cations (C_rel_) in solution, quantity of released cations per unit mass of the solid (Q_rel_), and quantity of residual cations on the solid (Q_res_) for the release of Y, Nd, and La from AC; Table S4: Concentration of released cations (C_rel_) in solution, quantity of released cations per unit mass of the solid (Q_rel_), and quantity of residual cations on the solid (Q_res_) for the release of Y, Nd, and La from MAC; Table S5: R^2^ values for the linear forms of Langmuir and Freundlich isotherms applied to the adsorption of Y, Nd, and La on AC; Table S6: R^2^ values for the linear forms of Langmuir and Freundlich isotherms applied to the release of Y, Nd, and La from AC; Table S7: R^2^ values for the linear forms of Langmuir and Freundlich isotherms applied to the adsorption of Y, Nd, and La on MAC; Table S8: R^2^ values for the linear forms of Langmuir and Freundlich isotherms applied to the release of Y, Nd, and La from MAC; Figure S1: (a) Adsorption efficiency (η_ads_) of Y, Nd, and La on AC; (b) Adsorption efficiency (η_ads_) of Y, Nd, and La on MAC; (c) Release efficiency (η_rel_) of Y, Nd, and La from AC; (d) Release efficiency (η_rel_) of Y, Nd, and La from MAC.
