# Supplementary material for: Analysis of the Adsorption-Release Isotherms of Pentaethylenehexamine-Modified Sorbents for Rare Earth Elements (Y, Nd, La)
Source: Polymers (Basel). 2022 Nov 22;14(23):5063. doi: 10.3390/polym14235063 (PMC9740061; doi:10.3390/polym14235063)
Supplement: Supplementary file 1 [file polymers-14-05063-s001.zip › polymers-1984454-supplementary.pdf]

Table S1. Initial concentrations (C_0), equilibrium concentrations (C_{eq}), and specific quantity of cations adsorbed on the solid (Q_{eq}) for the adsorption of Y, Nd, and La on AC.

C_0 [mmol L^{-1}]	Y		Nd		La	
	C_{eq} [mmol L^{-1}]	Q_{eq} [mmol g_{sol}^{-1}]	C_{eq} [mmol L^{-1}]	Q_{eq} [mmol g_{sol}^{-1}]	C_{eq} [mmol L^{-1}]	Q_{eq} [mmol g_{sol}^{-1}]
10	3.145	0.171	3.028	0.174	4.003	0.150
19	9.052	0.249	9.914	0.227	10.547	0.211
40	28.570	0.286	26.622	0.334	29.734	0.257
60	49.041	0.274	43.539	0.412	49.676	0.258
80	68.724	0.282	61.495	0.463	62.275	0.443
100	82.559	0.436	73.905	0.652	77.752	0.556
200	180.191	0.495	161.536	0.962	178.906	0.527

Table S2. Initial concentrations (C_0), equilibrium concentrations (C_{eq}), and specific quantity of cations adsorbed on the solid (Q_{eq}) for the adsorption of Y, Nd, and La on MAC.

C_0 [mmol L^{-1}]	Y		Nd		La	
	C_{eq} [mmol L^{-1}]	Q_{eq} [mmol g_{sol}^{-1}]	C_{eq} [mmol L^{-1}]	Q_{eq} [mmol g_{sol}^{-1}]	C_{eq} [mmol L^{-1}]	Q_{eq} [mmol g_{sol}^{-1}]
10	0.247	0.244	9.706E-5	0.250	0.006	0.250
19	4.892	0.353	3.367	0.391	–	–
40	22.316	0.442	17.540	0.562	18.653	0.534
60	40.515	0.487	35.981	0.600	34.556	0.636
80	56.802	0.580	57.750	0.556	52.552	0.686
100	73.112	0.672	76.815	0.580	68.751	0.781
200	152.071	1.198	150.441	1.239	171.340	0.716

Table S3. Concentration of released cations (C_{rel}) in solution, quantity of released cations per unit mass of the solid (Q_{rel}), and quantity of residual cations on the solid (Q_{res}) for the release of Y, Nd, and La from AC.

Y			Nd			La		
C_{rel} [mmol L^{-1}]	Q_{rel} [mmol g_{sol}^{-1}]	Q_{res} [mmol g_{sol}^{-1}]	C_{rel} [mmol L^{-1}]	Q_{rel} [mmol g_{sol}^{-1}]	Q_{res} [mmol g_{sol}^{-1}]	C_{rel} [mmol L^{-1}]	Q_{rel} [mmol g_{sol}^{-1}]	Q_{res} [mmol g_{sol}^{-1}]
3.546	0.136	0.035	3.227	0.124	0.050	2.710	0.104	0.046
4.204	0.162	0.087	4.475	0.172	0.055	3.463	0.133	0.078
4.753	0.183	0.103	5.675	0.218	0.116	4.063	0.156	0.101
5.031	0.194	0.080	5.671	0.218	0.194	4.541	0.175	0.083
5.196	0.200	0.082	6.652	0.256	0.207	5.759	0.222	0.221
7.731	0.297	0.139	7.141	0.274	0.378	6.954	0.267	0.289
8.886	0.342	0.153	8.527	0.328	0.634	9.539	0.366	0.161

Table S4. Concentration of released cations (C_{rel}) in solution, quantity of released cations per unit mass of the solid (Q_{rel}), and quantity of residual cations on the solid (Q_{res}) for the release of Y, Nd, and La from MAC.

Y			Nd			La		
C_{rel} [mmol L^{-1}]	Q_{rel} [mmol g_{sol}^{-1}]	Q_{res} [mmol g_{sol}^{-1}]	C_{rel} [mmol L^{-1}]	Q_{rel} [mmol g_{sol}^{-1}]	Q_{res} [mmol g_{sol}^{-1}]	C_{rel} [mmol L^{-1}]	Q_{rel} [mmol g_{sol}^{-1}]	Q_{res} [mmol g_{sol}^{-1}]
6.339	0.244	≈ 0	6.104	0.235	0.015	5.769	0.222	0.028
8.582	0.330	0.023	7.487	0.288	0.103	–	–	–
10.095	0.388	0.054	8.181	0.315	0.247	9.560	0.368	0.166
10.967	0.422	0.065	9.290	0.357	0.243	11.382	0.438	0.198
13.048	0.502	0.078	9.914	0.381	0.175	10.727	0.413	0.273

14.060	0.541	0.131	10.261	0.395	0.185	12.815	0.493	0.288
16.534	0.636	0.562	13.727	0.528	0.711	16.594	0.638	0.078

Table S5. R^2 values for the linear forms of Langmuir and Freundlich isotherms applied to the adsorption of Y, Nd, and La on AC.

**Linear form**	**R^2**		
	**Y**	**Nd**	**La**
Linear Langmuir 1	0.784	0.804	0.805
Linear Langmuir 2	0.902	0.804	0.895
Linear Langmuir 3	0.486	0.409	0.484
Linear Freundlich	0.818	0.939	0.845

Table S6. R^2 values for the linear forms of Langmuir and Freundlich isotherms applied to the release of Y, Nd, and La from AC.

**Linear form**	**R^2**		
	**Y**	**Nd**	**La**
Linear Langmuir 1	0.675	0.872	0.846
Linear Langmuir 2	0.125	0.751	0.009
Linear Langmuir 3	0.324	0.985	0.744
Linear Freundlich	0.751	0.885	0.689

Table S7. R^2 values for the linear forms of Langmuir and Freundlich isotherms applied to the adsorption of Y, Nd, and La on MAC.

**Linear form**	**R^2**		
	**Y**	**Nd**	**La**
Linear Langmuir 1	0.679	–	0.962
Linear Langmuir 2	0.770	0.735	0.996
Linear Langmuir 3	0.238	0.242	0.741
Linear Freundlich	0.807	0.671	0.963

Table S8. R^2 values for the linear forms of Langmuir and Freundlich isotherms applied to the release of Y, Nd, and La from MAC.

**Linear form**	**R^2**		
	**Y**	**Nd**	**La**
Linear Langmuir 1	0.878	0.644	0.670
Linear Langmuir 2	0.812	0.407	–
Linear Langmuir 3	0.996	0.935	–
Linear Freundlich	0.895	0.792	0.301

Figure S1. (a) Adsorption efficiency (η_{ads}) of Y, Nd, and La on AC; **(b)** Adsorption efficiency (η_{ads}) of Y, Nd, and La on MAC; **(c)** Release efficiency (η_{rel}) of Y, Nd, and La from AC; **(d)** Release efficiency (η_{rel}) of Y, Nd, and La from MAC.

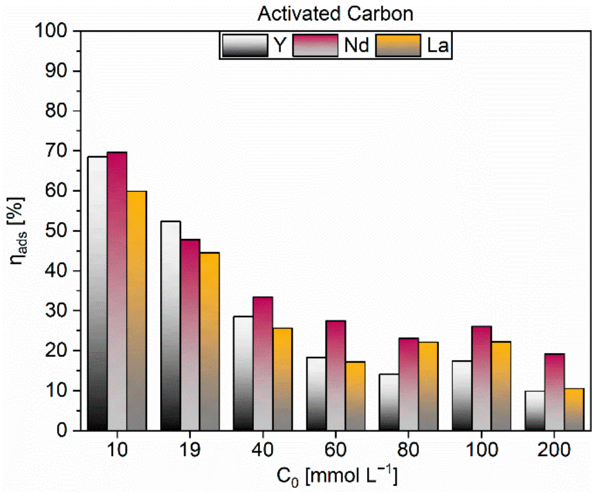

(a)

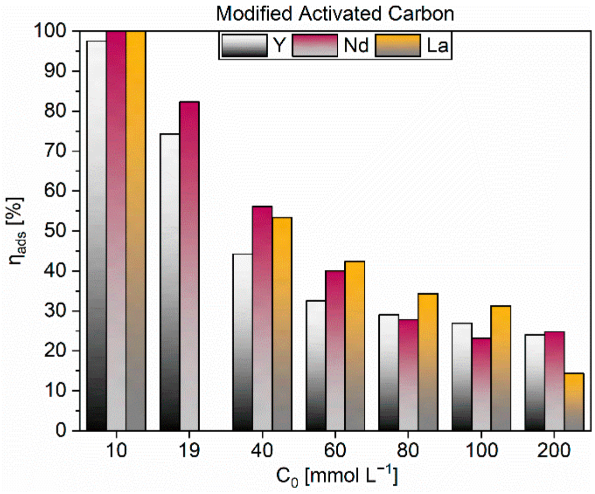

(b)

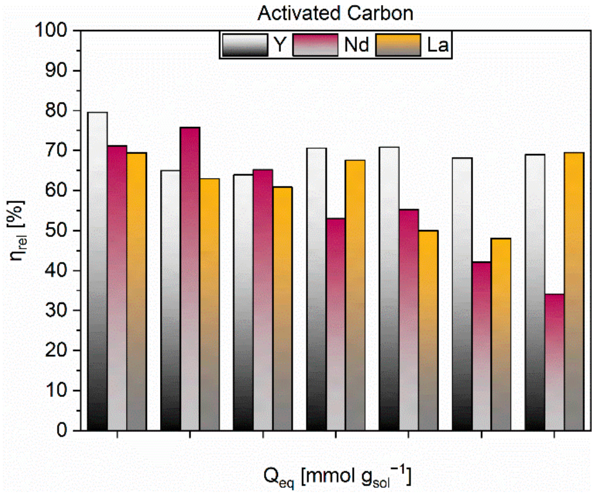

(c)

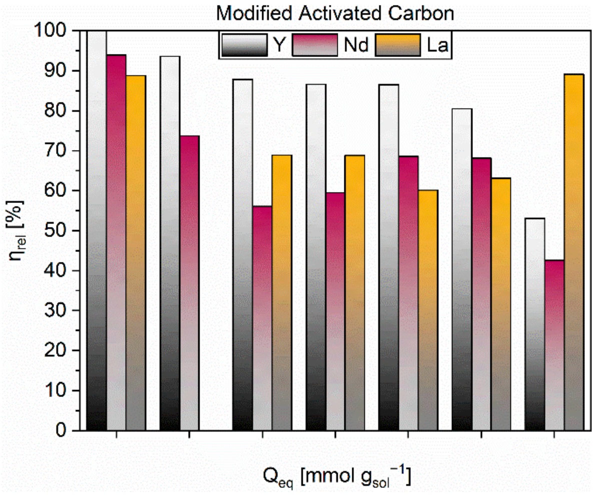

(d)
